# Full-Field Operational Modal Analysis of an Aircraft Composite Panel from the Dynamic Response in Multi-Impact Test

**DOI:** 10.3390/s21051602

**Published:** 2021-02-25

**Authors:** Ángel Molina-Viedma, Elías López-Alba, Luis Felipe-Sesé, Francisco Díaz

**Affiliations:** 1 Departamento de Ingeniería Mecánica y Minera, Campus Científico Tecnológico de Linares, Universidad de Jaén, 23700 Linares, Spain; lfelipe@ujaen.es; 2 Departamento de Ingeniería Mecánica y Minera, Campus Las Lagunillas, Universidad de Jaén, 23071 Jaén, Spain; elalba@ujaen.es (E.L.-A.); fdiaz@ujaen.es (F.D.)

**Keywords:** vision-based modal analysis, impact testing, aircraft structures, composite materials

## Abstract

Experimental characterization and validation of skin components in aircraft entails multiple evaluations (structural, aerodynamic, acoustic, etc.) and expensive campaigns. They require different rigs and equipment to perform the necessary tests. Two of the main dynamic characterizations include the energy absorption under impact forcing and the identification of modal parameters through the vibration response under any broadband excitation, which also includes impacts. This work exploits the response of a stiffened aircraft composite panel submitted to a multi-impact excitation, which is intended for impact and energy absorption analysis. Based on the high stiffness of composite materials, the study worked under the assumption that the global response to the multi-impact excitation is linear with small strains, neglecting the nonlinear behavior produced by local damage generation. Then, modal identification could be performed. The vibration after the impact was measured by high-speed 3D digital image correlation and employed for full-field operational modal analysis. Multiple modes were characterized in a wide spectrum, exploiting the advantages of the full-field noninvasive techniques. These results described a consistent modal behavior of the panel along with good indicators of mode separation given by the auto modal assurance criterion (Auto-MAC). Hence, it illustrates the possibility of performing these dynamic characterizations in a single test, offering additional information while reducing time and investment during the validation of these structures.

## 1. Introduction

Aircraft structures have to meet challenging requirements for performance and security and are thus subjected to strict controls and validation procedures. Experimentally, this involves a huge investment in complex facilities and equipment as well as long development and execution periods with multiple feedback iterations. During operation, they undergo multiple static and, especially, dynamic forces from different sources that have to be studied [1]. In this sense, modal analysis is essential in the linear dynamic characterization and validation of aircraft structures to understand their behavior under different operating conditions. This is commonly predicted by powerful numerical models using the finite element method [2] and estimated by challenging experimental instrumentations with several sensors [3]. The experimental study of the skin is particularly challenging considering the huge size of these structures. Therefore, instrumentations are commonly sparse so as not to increase the costs and complexity, and experimental modal analysis may yield spatial aliasing with misleading modal information [4].

Recently, new methodologies and techniques have been evaluated by different authors to perform full-field modal analysis that improves the spatial performance of the current instrumentation methodologies and provides a powerful tool for numerical model validation [5]. High-speed 3D digital image correlation (HS 3D-DIC) has achieved the highest relevance in this field. This is a noninvasive optical technique that provides 3D full-field displacement information from a surface which is monitored by a stereoscopic system composed of two high-speed cameras that perform high frame rates for dynamic events. Its capabilities for vibration measurement and modal analysis have been compared with other contactless alternatives such as scanning laser Doppler vibrometry [6,7,8]. These studies showed that 3D-DIC provides much higher spatial density and cost reduction for 3D measurement but its sensitivity is lower. However, it is also concluded that the lower sensitivity is mainly noticeable at high frequencies (of the order of a thousand hertz). Considering the advantages and limitations, mode shapes or operational deflection shapes take the greatest advantage among the modal parameters owing to high spatial density. Hence, many studies have employed this technique for simply obtaining shapes [9,10,11,12,13], even with low-speed equipment [14,15]. Full modal characterization has been also performed in different studies for experimental modal analysis [16,17,18], showing different capabilities and applications such as the combination of measurements with a multiview system [19], the evaluation of a single-camera alternative to DIC for 3D measurements [20] or measuring in rotating disks [21] and in artificial beetle’s wings [22]. Furthermore, the technique has shown potential for vibration-based methodologies for structural health monitoring [23], evaluating impact damage severities on mode shapes [24] or locating damages taking advantage of the dense information [25].

Most of these studies evaluated the bending modal behavior of plate-like elements as the technique is quite appropriate for wide and thin flexible elements. Some of them are aircraft structural elements [13,26,27]. The skin is typically made out of composite materials, which significantly improves the strength with an important weight reduction. However, ductility is much lower than metals and brittle fracture may occur. Brittleness is concerning under impacts since it may produce penetration or cracks affecting the air-tightness and security of the crew members and passengers. These forces are present during operation in the form of hail, birds or unidentified objects behaving as projectiles. Experimentally, brittleness and energy absorption have been evaluated using different methodologies and facilities. For low-velocity impacts, drop-weight tests are typically performed [28]. Conversely, high-velocity impacts have been commonly employed with light-weight projectiles such as ballistic bullets [29,30] or using gas gun facilities that allow the use of different kinds and shapes of projectiles [31,32,33].

Although the characterization of the impact performance in terms of brittleness and energy absorption is typically evaluated under a single projectile impact, a more realistic characterization of the degradation and cumulative damage can be done using multihitting. Two scenarios can be considered here, depending on whether the stress-wave interaction of each projectile impact occurs or not: simultaneous multi-impact or sequential impact, respectively. The next works are good examples of that. The effect of strength and ductility under sequential impacts was studied by Russel in stainless steel plates [34]. The response of laminated composites under three simultaneous impacts using a gas gun was investigated by Deka et al. [35]. In addition, Garzon-Hernandez et al. studied the effect of single, sequential and simultaneous impacts on polyether-ether-ketone (PEEK) reinforced with short carbon fiber [36].

The dynamic response after an impact is the resulting vibration of a broadband excitation [3,4]. Traditionally, the vibration response to low-energy impacts using an instrumented hammer with a force sensor has been measured by HS 3D-DIC to perform experimental modal analysis under laboratory conditions [16,17,19]. Likewise, the vibration response of the panel after simultaneous multi-impact can be exploited to extract modal parameters. Despite the high velocity of the projectiles and the brittle behavior of composite materials such as carbon-fiber-reinforced polymer (CFRP), they show high stiffness. Thus, no large displacements are expected during the vibration that may induce relevant nonlinearity. Besides, nonlinearity due to damage generation mechanisms can be neglected as long as nonsevere damage or penetration occurs.

However, the multihitting entails unknown force conditions owing to the difficulty of describing the force transmitted by every projectile and the location of every single impact. Therefore, operational modal analysis (OMA) has to be considered, which is a response-only methodology that assumes that the spectral excitation level is constant in the band of interest. Previous studies have explored OMA on HS 3D-DIC measurement. In [37], the authors performed the first integration of DIC and OMA for the analysis of a helicopter rotor blade during operation using the Ibrahim time-domain method. Considering the amount of data generated by full-field measurement, the evaluation of a compressed DIC sensing methodology has been performed for OMA using the stochastic subspace identification time-domain method [38,39]. In [40], the authors performed an OMA of the flexible blades of a two-bladed rotor using DIC measurements. For the modal identification, a combination of natural excitation technique and eigensystem realization algorithm was performed and compared with the complexity pursuit method. In [41], the authors employed the mode shapes extracted by Bayesian OMA with HS 3D-DIC to locate damages in membranes.

In this work, the possibility of performing modal identification from a high-velocity simultaneous multi-impact test with a gas gun, intended for impact characterization, is explored to offer a wide dynamic characterization from a unique test in composite skins that can be employed for multiple purposes like model validation or damage detection. HS 3D-DIC was employed to measure the full-field displacement response of a CFRP aeronautical panel to such impact excitation, with unknown quantitative information of the excitation. The full-field information was employed for OMA using the polyreference least-squares complex frequency-domain method (PolyMAX) to estimate the modal parameters. The analysis succeeded in obtaining the modes with good quality indicators.

## 2. Experimental Methodology and Analysis

### 2.1. Specimen Description

The structural test was conducted on a specimen of a stiffened composite panel representative of cockpit fuselage skin for a regional aircraft. This panel was designed, manufactured and tested by Airbus as part of the Clean Sky program [42], of which the University of Jaén took part through a contactless measurement using high-speed cameras and 3D-DIC during multi-impact testing.

**Figure 1:**
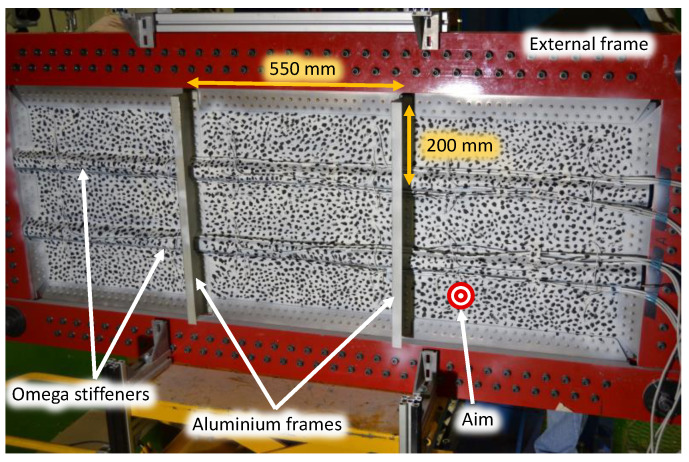
Carbon-fiber-reinforced polymer (CFRP) aeronautical panel employed to evaluate its modal behavior after multi-impact excitation using high-speed 3D digital image correlation (HS 3D-DIC).

The specimen was a 2.3 mm thick CFRP aeronautical panel, shown in Figure 1, stiffened with two horizontal CFRP omega stiffeners of 1 mm thickness and two vertical aluminum frames [43]. The dimensions of the entire test panel and each bay were 600 mm by 1655 and 200 mm by 550 mm, respectively. It was rigidly framed in a metallic structure using a staggered bolt union. The cables observed on the specimen surface were installed for purposes not related to the current test.

### 2.2. Multihit Test Setup

During the test, the panel was impacted on its nonstiffened surface with multiple polymeric spherical projectiles (12.6 mm in diameter and 1.3 g) at 50 m/s using a gas gun. The projectiles were shot in a gathered way aiming for the lower right bay of the panel, as highlighted in Figure 1. These projectiles in laboratory conditions allow the reproduction of real multi-impacts on the panel during operation such as hail. The whole system was supported by a tray and slings to reduce the motion of the structure after the impact. The low mass of the projectiles in contrast to the rig made the impacts mainly produce deformation in the panel with a negligible motion of the whole rig. In addition, this subtle effect is easily subtracted by identifying the rigid body motion component in the displacement maps.

The projectiles were expected not to impact at the same time instant. However, the lag between the first and last one (of the order of 10 µs [36]) is many times lower than the vibration period in the band of interest. Therefore, the multihit can be considered as a unique impact for this purpose. The demonstration is addressed in Section 3. Likewise, the impact locations of every projectile, although close to each other, are different. However, this circumstance was initially considered as a multi-input excitation test. In conclusion, the lack of force signal and location leads to performing an OMA.

### 2.3. Time Series DIC Measurement

**Figure 2:**
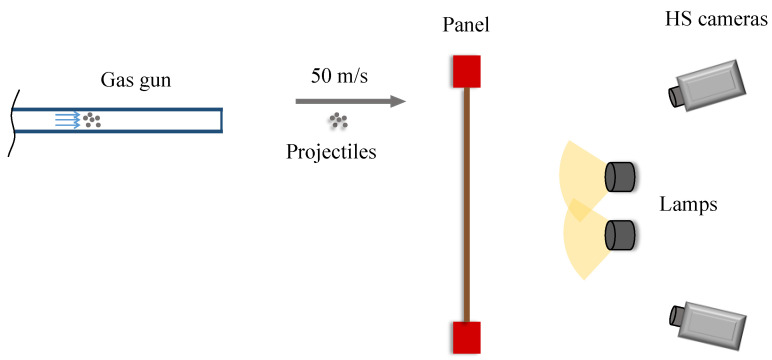
Schematic of the experimental setup for HS 3D-DIC measurement of the vibration response of a CFRP panel to multi-impact excitation using a gas gun.

The response to such impacts was monitored on the other side of the impacted surface by two high-speed cameras (Photron SA4) to provide stereovision, as shown in Figure 2. The cameras recorded 4500 frames per second (1024 × 896 pixels) with 50 mm f/1.4D focal length Nikon lenses. This was the maximum frame rate available after cropping the 1 Mpixel squared image to fit the panel. The cameras were placed in front of the rear surface of the panel to be monitored with a stereo-angle of 28.3°, according to the calibration parameters. Once the projectiles are shot, they move quickly and cover the distance to the panel in a very short time. In order to make the cameras accurately record just the impact event, taking the most advantage of the image sequence, a trigger signal commanded the cameras to start recording immediately before the impact occurred. This was achieved by a signal sent from an optical sensor placed in the gun that detected the projectiles. In this way, it was possible to record with a very short pre-impact-measurement time lapse.

Good contrast of the speckle is one of the most important parameters for achieving a successful and accurate correlation. Generally, ambient light conditions are not enough to perform satisfactory measurements, but, especially, high-speed recording reduces the light received by the sensor and produces very dark images. During this test, two light sources were employed to achieve a homogeneous light intensity on the panel. The whole setup is illustrated in a schematic arrangement in Figure 2. The observed surface was coated with white paint, and the final speckle pattern was generated by superimposing black paint dots in random distribution, shape and size but according to the image resolution (about 6 pixels in diameter on average).

**Figure 3:**
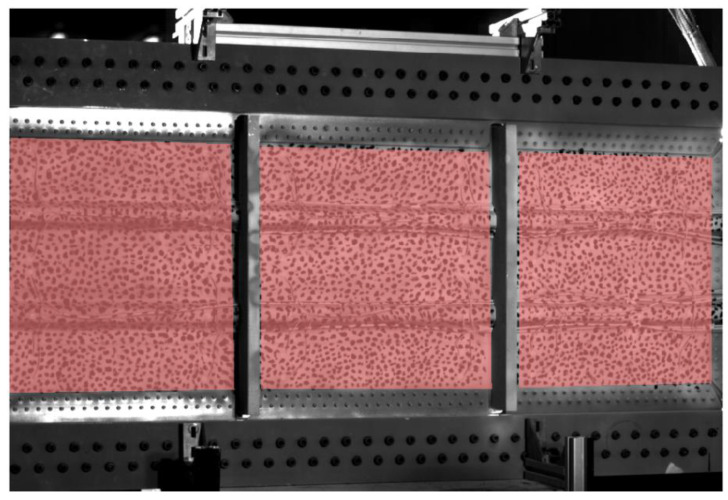
Regions of interest for DIC measurement in the composite panel.

The resulting images were afterward processed through 3D-DIC to obtain quantified displacement fields. The processing was performed in VIC 3D software by Correlated Solutions. The analysis included the omega stiffeners but not the aluminum frames owing to the larger difference in depth. These aluminum frames were also a physical obstacle for the vision of the cameras. These issues compromised the correlation area, and hence the region of interest was originally divided into three masks to optimize the correlation around the frames. The correlation analysis was performed using the same parameter for every mask, namely facets of 21 pixels and 3-pixel spacing between the central pixels of adjacent facets. A small step was chosen to improve the spatial resolution in narrow areas such as the omegas. The region of interest is shown in Figure 3 with a grid of contiguous facets overlaid.

**Figure 4:**
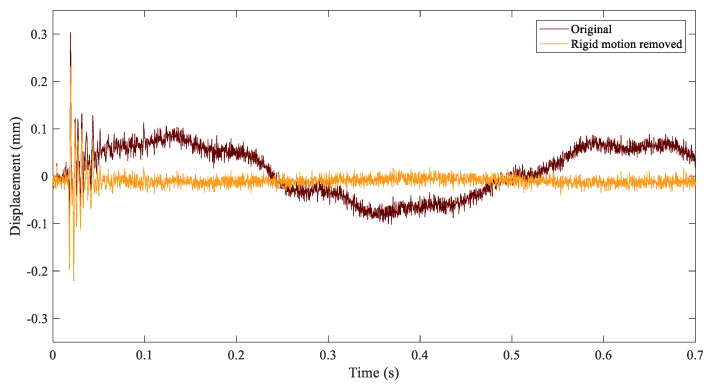
Raw out-of-plane displacement evolution of a point in the middle of the panel and the resulting evolution after removing the rigid body motion.

Bending deformation is the consequence of the perpendicular impacts in the panel. Therefore, in-plane deformation was neglected and only out-of-plane displacements, perpendicular to the plate, were considered for analysis. During the inspection of the time series, such as in Figure 4, a large period wave was identified as the rigid oscillation of the panel over the expected higher frequency vibration of the panel deformation. This rigid motion was removed using a subroutine in VIC software. The method calculates an average transformation for each image and inverts it to obtain an image with a zero-average displacement/rotation [44]. The result of the signal before and after the solid rigid motion was removed is illustrated in Figure 4. After the removal of solid rigid motion, the analyzed displacements involved pure panel deformation.

### 2.4. Operational Modal Analysis

The results were then processed using LMS operational PolyMAX for OMA, which is a polyreference version of the least-squares complex frequency-domain method and is known to produce very clear stabilization diagrams [45]. For the calculation of the cross-power spectra, only the first 700 images, i.e., time instants, were employed, as this window contains the displacement evolution until complete dissipation. Hence, no exponential window correction was required to reduce leakage. This test has some particularities differing from a laboratory-condition impact hammer test that make it unfeasible to reproduce it repeatedly. One issue would be the difficulty to shoot projectiles to an identical location during multiple tests. Moreover, repeating such a severe impact would affect global behavior as significant cumulative damage could appear. Thus, it was not possible to repeat the excitation to perform averaging processing in the frequency domain to reduce the noise in the measurement. Instead, this test supplied much more energy in a single impact to produce higher perturbation and less noisy measurement than a hammer would have produced in a panel of that size and stiffness. Previous studies performing modal identification with DIC measurement in impact hammer tests were performed on light and flexible components with low constraints (typically under free–free boundary conditions) [12,17,27,46] in contrast to this panel, with stiffeners and a metallic frame constraining the boundaries.

To check the linear independence of the mode shapes, the auto modal assurance criterion (Auto-MAC) [47] was employed. This is a variant of MAC, which shows the degree of consistency between two modal vectors, {*ψ_i_*} and {*ψ_j_*}, applied to the set of mode shapes of a single estimation:

(1) $$MAC_{ij}=\frac{\left|{\left\{\psi_{i}\right\}}^{H}\left\{\psi_{j}\right\}\right|}{{\left\{\psi_{i}\right\}}^{H}\left\{\psi_{i}\right\}{\left\{\psi_{j}\right\}}^{H}\left\{\psi_{j}\right\}}$$

The superscript ***H*** indicates the Hermitian complex conjugate transpose. The MAC takes values from 0 to 1 depending on the consistency of the evaluated modal vectors. A high level of consistency would produce a value close to 1 and vice versa. Considering a single set of modes, a low value of Auto-MAC would indicate linear independence. Hence, when the Auto-MAC of a modal vector against itself is determined, it takes the value of 1.

## 3. Results

**Figure 5:**
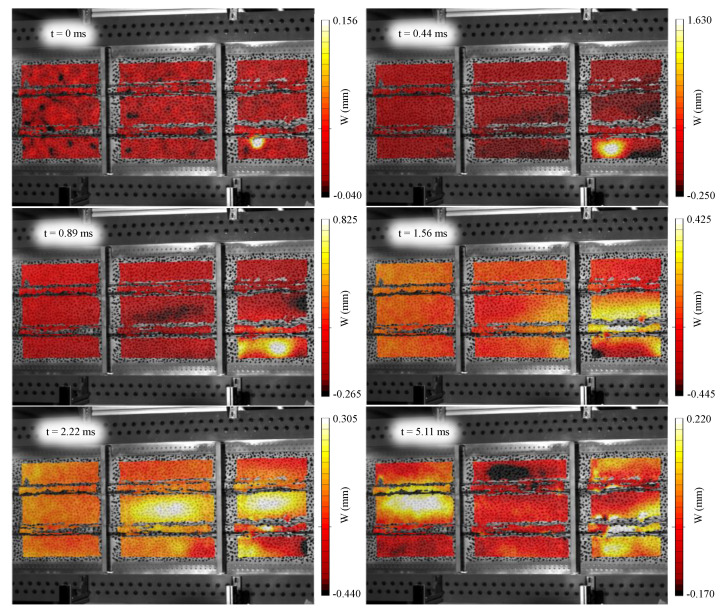
Evolution of the out-of-plane displacements after the impact at different time instants with respect to the very first contact.

**Figure 6:**
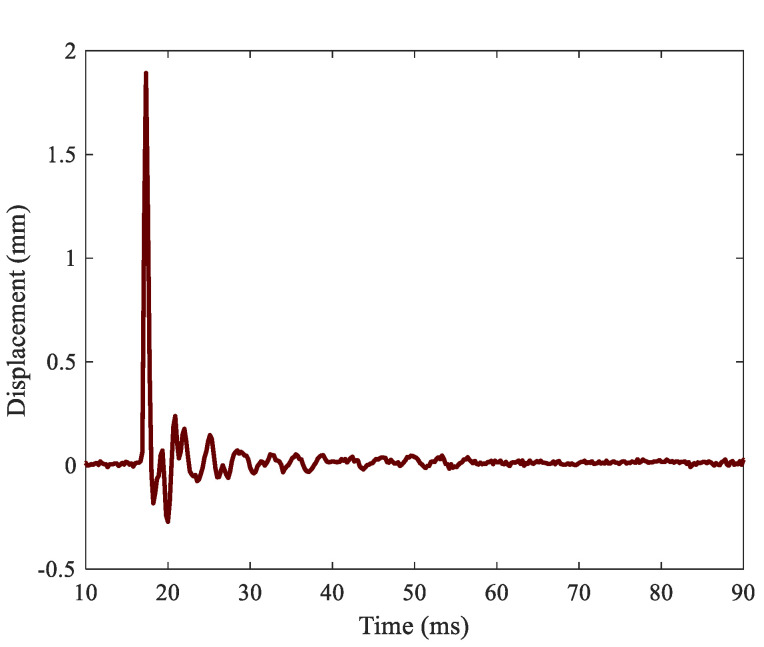
Vibration response at a point in the impacted area in absolute time after the start of recording. Simultaneous multihits are noticed as only one peak.

As a result of the test previously described, a time series of displacement maps was obtained concerning the full event. Figure 5 shows six different time instants relative to the first image where the impact was detected. After an initial wave propagation observed in the first four images, the panel vibrated randomly with predominance of its resonances. As stated above, each projectile impacted the panel at different instants, but the lags were supposed to be insignificant in this study. This fact can be observed in the results. Observing the displacement maps at different time instants, only one impact was identified at 0.44 ms after the first contact, as seen in Figure 5. Focusing specifically on the impacted region, the absolute time history at the maximum deformation point, in Figure 6, does not show any additional peak besides the vibration response. All this confirmed that the multihitting can be considered simultaneous in comparison with the time-scale vibration response and much faster than the sampling frequency. From Figure 5 and Figure 6, it is also possible to infer the maximum displacement in the panel quantified as 1.893 mm, corresponding to the time instant 0.44 ms relative to the first contact. Afterward, a drastic amplitude reduction follows. According to the panel size, the maximum displacement is of the order of 100 times lower, and small strains can be thus assumed. Overall, a signal-to-noise ratio of 26.1 on average was reached considering every measurement point in the panel.

**Figure 7:**
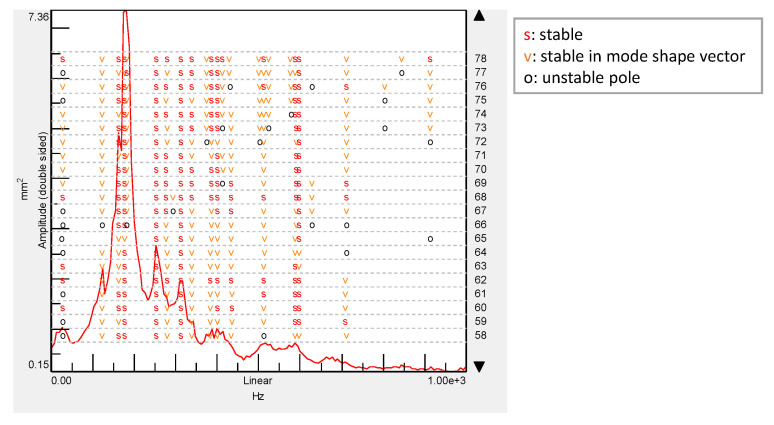
Stabilization diagram obtained by applying LMS operational polyreference least-squares complex frequency-domain method (PolyMAX).

**Figure 8:**
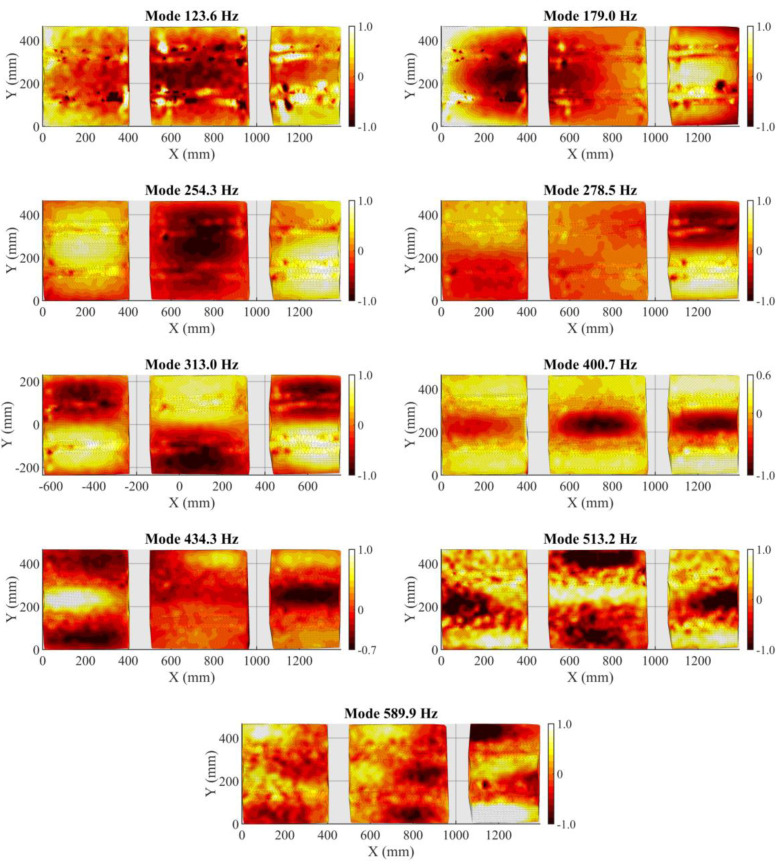
Full-field mode shapes of the CFRP aeronautical panel using OMA.

After this first inspection, the full-field time histories were gathered and prepared for OMA. The mode identification was initially performed through the stabilization procedure. The stability of the resonances as the model order increases is an indicator of the poles of the system. Figure 7 shows the stabilization diagram up to a model order of 78 over the sum of the displacement cross-power spectra. The poles found in the system are indicated in this plot, which highlights those that provide stable modal parameters as the order of the model increases. Nine stable poles that only represent modes of the panel were selected to construct the model of the panel. Computational modes, or those induced by other accessories on the surface such as cable motion (sensors installed for a different study), were excluded after observing the mode shapes and incoherent damping ratios. The corresponding natural frequencies and damping ratios are listed in Table 1, and the mode shapes are shown in Figure 8 using amplitude normalization. For the 3D spatial illustration of mode shapes, Delaunay triangulation was employed. Every mode shows remarkable complex bending modes considering the stiffness of the panel, but none of them show a local deformation around the impacted region to be considered affected by the possible damage. As can be observed, the shapes are very influenced by the omega stiffeners and the frames. This does not occur for the lowest order modes, as the bending areas between nodes of zero displacement are longer than the bays. Moreover, different perturbations were observed nearby the omega stiffeners in the lowest frequencies. They are a consequence of the cables that vibrated over the speckle pattern and induced, in this way, local errors in the correlation. This issue has low impact on the interpretation of the actual mode and does not alter the purpose of this study. In some modes, damping is low (less than 1%), which might be due to the intrinsic assumptions of an OMA. This is especially the case for the two highest modes where the level of displacement is lower and therefore closer to the DIC noise floor. Under this consideration, the highest mode (589.9 Hz) tends to show a second-order bending shape in the middle bays, but the displacement was too subtle to extract meaningful information in this case. This is one of the restrictions for HS DIC or any camera-based technique in general for vibration and modal analysis. As displacement amplitude typically decreases as the frequency increases, these techniques for displacement measurement provide better results for low frequencies. The maximum frequency that is feasible to analyze depends on the specimen, the type and amplitude of excitation, the quality of the speckle, the resolution of the camera and the overall setup, ranging from hundreds to thousands of hertz [11,48,49]. Therefore, above this last resonance frequency, the method was not able to perform any modal identification with a minimum stability or trace of actual behavior. This was also the reason for limiting the analyzed spectrum during the modal identification. Considering this, the fact that the first mode appears slightly noisier than subsequent modes does not seem to support this argument. However, it is possible to observe that the impact was very close to a nodal region (zero deformation) of this mode and, consequently, generated lower response [3]. For the same reason, its damping ratio should be carefully considered.

**Figure 9:**
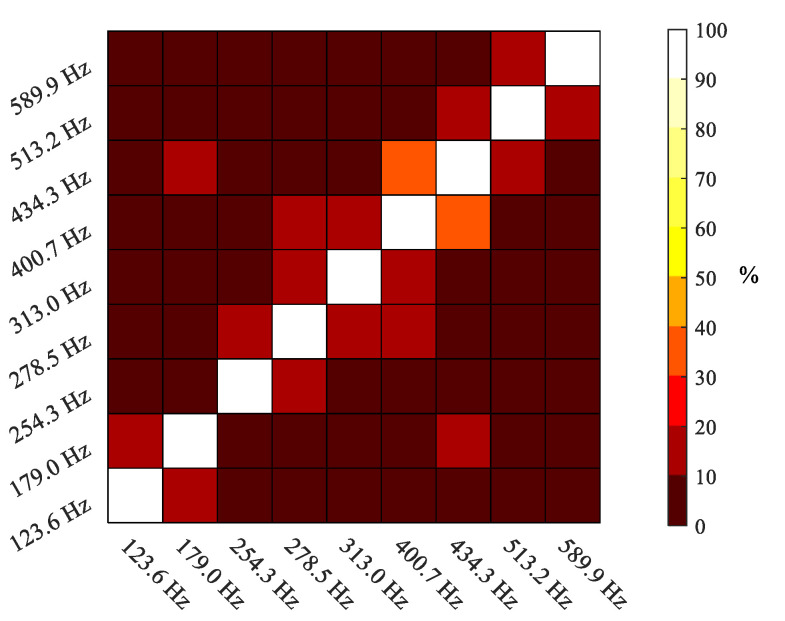
Auto modal assurance criterion (Auto-MAC) of the obtained modal model.

The analysis of these results ends with checking the quality and linear independence of the set of mode shapes through the Auto-MAC, according to Equation (1). The result is presented in Figure 9. The matrix of correlation coefficients reveals a very low correlation between nonidentical mode shapes, out of the diagonal, and just close to 30% for correlative modes. Although there was no chance to perform a validation measurement, the Auto-MAC results allow confirming that the extracted modes are physically different and the model is close to orthogonality. Moreover, the validity of HS DIC measurement for vibration and modal analysis has been thoroughly proven in the literature [6,11,17].

## 4. Conclusions

The aircraft industry makes remarkable efforts to increase the efficiency of testing procedures during the development of structures and complex materials to reduce time and costs. This study presents an innovative alternative by including modal identification in impact behavior characterization performed in simultaneous multi-impact testing, originally intended for studying the effect of hail, birds or other unidentified projectiles. Hence, it takes advantage of this complex setup and expensive facilities. This has been shown through an operational modal analysis in an aeronautical composite panel using the response to a high-velocity simultaneous multi-impact test measured by HS 3D-DIC in a full-field manner, assuming linear conditions. In particular, the small strain condition was checked through the level of displacement after the impact. Despite the challenging conditions, the operational modal analysis provided the natural frequencies, damping ratios and full-field mode shapes of different modes of the panel up to 600 Hz. The modal parameters were coherent with the multiple order bending deformation of a plate-like structure, also influenced by the stiffener configuration of the panel. Furthermore, Auto-MAC indicated independence of the obtained shapes. Therefore, a unique test rig can be employed for impact analysis and modal characterization under valid assumptions. Additionally, this combination may offer new possibilities in future studies such as evaluating the cumulative damage produced by multiple simultaneous multi-impacts using traditional local damage assessment methodologies and also monitoring the global effect on the modal parameters for structural health monitoring.

## Figures and Tables

**Table 1:** Natural frequency and damping ratio of the CFRP aeronautical panel using operational modal analysis (OMA).

Natural Frequency (Hz)	Damping Ratio (%)
123.6	0.89
179.0	3.07
254.3	2.62
278.5	2.15
313.0	1.78
400.7	0.82
434.3	0.88
513.2	0.18
589.9	0.61

## Data Availability

Not applicable.
